# Examining the interplay of childhood abuse, bullying, and bullying victimization in middle school students: a serial mediation analysis

**DOI:** 10.3389/fpsyg.2025.1586797

**Published:** 2025-08-06

**Authors:** Ting Bai, Min Lang, Yue Jin, Jingyi Li, Peng Chen

**Affiliations:** ^1^School of Education and Psychology, Sichuan University of Science and Engineering, Zigong, China; ^2^School of Education and Psychology, Chengdu Normal University, Chengdu, China; ^3^Zigong Fourth People’s Hospital, Zigong, China

**Keywords:** bullying, bullying victimization, childhood abuse, self-esteem, peer relationships, moral disengagement

## Abstract

**Introduction:**

Childhood abuse and bullying are youth problem worldwide, yet the detrimental effects of child abuse is underestimated in China. The aim of this study is to analyze the relationship between child abuse and bullying and victimization, and to explore the mediating roles of self-esteem, peer relationships, and moral disengagement in the chain between childhood abuse, school bullying, and victimization among junior high school.

**Methods:**

This study included 1,327 adolescents (52.3% girls, 47.3% boys) from public schools in Sichuan Province, China. We conducted a cross-sectional questionnaire survey using the Child Trauma Scale-Short Form, Bullying Participatory Behavior Questionnaire, Self-Esteem Scale, Peer Relationships Scale, and the Moral Disengagement Questionnaire. The bootstrap technique was used to conduct mediation analysis. SPSS Process Macro 3.0 control prepared by Hayes, Gender and grade were used as the control variables in model 6.

**Results:**

Childhood abuse significantly predicts both bullying (B =0.08, *p* < 0.001) and victimization (B =0.17, *p* < 0.001). Meanwhile, the serial mediating effect via self-esteem, peer relationships and moral disengagement between childhood abuse and bullying was 0.0015 (95% CI: 0.0003–0.0029), the serial mediating effect via self-esteem, peer relationships and moral disengagement between childhood abuse and bullying victimization was 0.0005 (95% CI: 0.0000–0.0014).

**Discussion:**

The investigation into the chain mediating effects of self-esteem, peer relationships, and moral disengagement on the interconnections between childhood abuse, bullying, and bullying victimization has deepened our understanding of the cycles of violence and victimization. It has pointed out that family factors may be key elements in bullying prevention.

## 1 Introduction

Research on school bullying began with empirical studies in Scandinavia in the 1970s (Olweus, 1978). Despite the constantly changing forms of school bullying, it remains a hot research topic. This ongoing attention is rooted in social concern for the healthy growth of adolescents, reflection on the need for a safe living environment in schools, and the threat of violence not diminishing but rather becoming increasingly intense with the development of society. Bullying is defined as a subcategory of interpersonal aggressive behavior, characterized by intentionality, repetition, and a power imbalance, with the abuse of power being the primary feature that distinguishes bullying from other aggressive behaviors (Kanetsuna et al., 2006). Salmivalli et al. (1998) categorized the roles in school bullying participation behaviors into victim, bully, reinforcer of the bully, assistant of the bully, defender of the victim, and outsider. Bullying behavior not only severely affects the physical and mental health of young individuals (Graham, 2016) but also hinders individual growth and development and may even induce antisocial behavior (Sindhu et al., 2024). Moreover, bullying has a profound impact on the school’s safe environment (Yang et al., 2021), with both bullies and victims presenting a significant threat to the safety of the school environment (Dorio et al., 2019).

Child abuse is a pervasive global issue with a highly variable prevalence rate, ranging from 5% to 25% overall. Specifically, physical abuse prevalence varies from 2% to 78%, sexual abuse from 2% to 47%, and emotional/psychological abuse from 2% to 69% (Brunton, 2024). According to the cycle of violence, or intergenerational transmission of violence, theory, adolescents who have experienced childhood abuse are more likely to inflict the harm they have suffered on others, thus becoming bullies (Widom, 1989). The long-term negative effects of experiencing childhood abuse, such as emotional problems (Arslan, 2016), cognitive dysfunction (Su et al., 2019), self-harm, and suicidal behavior (Macalli et al., 2021), make individuals more likely to become targets of bullying, forming a cycle of victimization (Widom, 2013).

Therefore, the relationship between childhood abuse and bullying, as well as bullying victimization, is a matter of concern. Moral disengagement is a cognitive factor that acts as a bridge between them (Ding et al., 2022). A negative correlation exists between self-esteem and the negative consequences of childhood abuse (Arslan, 2016; Díaz-Faes and Widom, 2024), and peer relationships predict these negative consequences (Tung et al., 2018; Yoon et al., 2019; Yoon et al., 2021).

However, research that integrates family, personality, peer, and cognitive factors to explore the mechanisms behind bullying behavior is lacking. Analyzing bullying behavior could help develop interventions to prevent school bullying, and the impact of childhood abuse on bullying through multiple factors must be explored. This study aims to explore how childhood abuse impacts bullying and victimization, and the roles of self-esteem, peer relationships, and moral disengagement in this context. Thus, this study aims to address the following research questions: First, examine the predictive role of childhood abuse on bullying and bullying victimization. Second, examine the serial mediating effect of self-esteem, peer relationships, and moral disengagement between childhood abuse and bullying. Third, evaluate the serial mediating effect of self-esteem, peer relationships, and moral disengagement between childhood abuse and bullying victimization.

## 2 Literature review

### 2.1 Childhood abuse and bullying

The World Health Organization (1999) defined childhood abuse as actions by individuals who have the care, supervision, and control of children that can cause actual or potential harm to the child’s health, survival, growth, and dignity, including physical abuse, emotional abuse, sexual abuse, neglect, and deprivation.

The occurrence of bullying behavior often has no clear cause and can be considered a form of abuse. Stratford (1996) and others have defined bullying behavior as systematic power abuse. Power relationships are formed in social groups through strength, ability, personality, hierarchy, and status. Whether an action constitutes power abuse depends on the specific social and cultural environment. Systematic, repeated, and intentional power abuse is considered bullying. The bully is the person who inflicts violence on the victim, and the victim is the target of physical, verbal, relational, or cyberbullying (Taki, 2009). Research has shown that parent–child relationship variables, such as child abuse and neglect, can affect bullying and bullying victimization among children (Li et al., 2024).

Childhood abuse or neglect increases the risk of individual criminal behavior and activity (Chen et al., 2024; Heirigs, 2021). Children who have been abused are more likely to bully other children than those who have not been abused, leading to the transmission of violence (Papalia et al., 2025). Furthermore, children who have been abused are nearly two times more likely to engage in criminal activities and continue criminal behavior than other children (Braga et al., 2018) and three times more likely to experience psychopathology than those who have not been abused (Russotti et al., 2021). Abused children are likely to have difficulty regulating their emotions (Albert, 2017), more prone to anger, and more aggressive than those who have not been abused (Keene and Epps, 2016). According to the social learning theory, children who suffer abuse from people around them gradually learn the same method of dealing with the world, that is, fighting fire with fire (Albert, 2017). Most participants in bullying behavior have a history of childhood abuse (Li et al., 2024; Zhang et al., 2023). Theoretical and practical evidence has identified a direct relationship between childhood abuse and bullying behavior. Therefore, we propose H1a: childhood abuse is positively correlated with bullying behavior.

### 2.2 Childhood abuse and bullying victimization

The developmental cascades theory posits that human development is a process in which multiple developmental characteristics continuously interact in a cascading manner (Masten et al., 2005). At a certain point in time, the developmental state of an individual characteristic affects the subsequent development of that characteristic. The cumulative interaction or interplay of various factors may lead to propagation effects across various levels, domains, systems, and even generations (Masten and Cicchetti, 2010). Widom (2013) suggested the existence of a cycle of victimization, in which victims of abuse and neglect may be re-victimized in their future lives. Therefore, early traumatic experiences are likely to result in continued victimization in other settings later in life. The learned helplessness created by childhood trauma experiences causes a lack of resistance when these children are subjected to bullying, thus becoming long-term victims of it (Peterson and Seligman, 1983). Furthermore, from a cognitive perspective, children who have experienced childhood trauma are more likely to be unpopular than those who have not experienced it, making them targets of bullying (Su et al., 2019). Therefore, we hypothesize that Childhood abuse positively predicts bullying victimization (H1b).

### 2.3 The mediating role of self-esteem

Self-esteem refers to an individual’s self-assessment of their social roles and can affect the development of their behavior (Rosenberg, 1965b). The adverse consequences of childhood abuse are the emergence of emotional and behavioral problems (Russotti et al., 2021; Yoon et al., 2021), which are closely related to the occurrence of low self-esteem (Arslan, 2016). Experiences of abuse can predict an individual’s level of self-esteem, with children who have been abused often exhibiting low self-esteem (Oshri et al., 2016). Individuals with low self-esteem tend to have excessively negative self-assessments and be more aggressive and prone to feelings of frustration and anger than those with high self-esteem (Teng et al., 2015). When negative emotions, such as anger and hostility, are not properly managed, reactive aggression is likely to occur (Amad et al., 2020). Therefore, low self-esteem resulting from experiences of childhood abuse may lead to bullying behavior. We hypothesize that self-esteem mediates the relationship between the two variables (H2a).

Self-esteem has a predictive effect on bullying victimization (Touloupis and Athanasiades, 2022). People with low self-esteem are more likely to become the targets of aggression or bullying in their interactions with others than those with high self-esteem (Agustiningsih et al., 2024). The self-perception driven model argues that self-esteem has an indirect impact on peer victimization. Therefore, we hypothesized that self-esteem would mediate the relationship between childhood abuse and bullying victimization (H2b).

### 2.4 The mediating role of peer relationship

The types of childhood abuse can lead to differences in peer relationship (Yoon, 2020; Yoon et al., 2021). Moreover, parent–child relationship influences the quality of children’s peer relationship (Zhang and Deng, 2022; Zhou et al., 2023). Studies on peer dynamics, including likeability, rejection, friendship, and acceptance, have found that children who have suffered abuse exhibit higher levels of maladaptation in peer relationships than those who have not (Yoon et al., 2018; Tung et al., 2018).

Specifically, experiencing physical, sexual, and emotional abuse increases the risk of re-victimization during adolescence (Benedini et al., 2016). Furthermore, experiencing high levels of peer rejection is associated with externalizing behavior problems, such as violence and aggressive behavior (Yoon et al., 2021). In addition, deviant peer affiliation directly predicts bullying and bullying victimization (Liang et al., 2022).

Peer popularity is associated with the adverse behavior of maltreated children and adolescents (Yoon, 2020). Bullying is often used as a means to gain peer status, with high-status adolescents being more popular among their peers and having greater influence than low-status ones (Vitaro et al., 2007). Conversely, a low peer status increases the risk of bullying victimization (Zhang et al., 2018). Therefore, we hypothesized that early experiences of abuse would lead to bullying (H3a) or bullying victimization (H3b) through peer relationships.

### 2.5 The mediating role of moral disengagement

Abuse experiences significantly increase an individual’s level of moral disengagement (Fang et al., 2020). Bullying is essentially a moral issue. Moral disengagement is the internal psychological mechanism that affects school bullying. It allows individuals to commit immoral actions without feeling guilty or self-blaming (Gini, 2006). Strong moral disengagement is associated with greater bullying tendencies and behaviors (Gini et al., 2011). Therefore, we hypothesized that experiences of abuse would affect bullying behavior through moral disengagement.

Individuals who have been abused may cope with the injustice and harm they have experienced through moral disengagement, thereby reducing their feelings of guilt and psychological stress (Wang et al., 2017). The mechanism of moral disengagement helps explain involvement in school bullying, including both bullying and bullying victimization (Wang et al., 2022). We hypothesized that moral disengagement would mediate the relationships between childhood abuse, bullying (H4a) and bullying victimization (H4b).

### 2.6 The present study

Existing research has confirmed the relationship between childhood abuse and bullying and victimization, and has verified the existence of the cycle of violence and the cycle of victimization. However, the pathways from childhood abuse to bullying and victimization have not been fully explored. The personality, environmental, and cognitive factors that influence the cycle of violence and the cycle of victimization have not been adequately explained.

#### 2.6.1 The serial mediating role of self-esteem and peer relationships

The attachment theory of self-esteem posits that self-concept and self-worth develop through repeated interactions with significant others, individuals who grow up in environments of neglect and abuse do not receive adequate care and attention, making it difficult for them to form secure attachment relationships and likely to develop low self-esteem (Oshri et al., 2016). Low self-esteem, in turn, hinders the establishment of attachment relationships with peers, leading to poor peer relationships. According to the general strain theory, when individuals experience external stress (poor peer relationships), they may resort to aggressive methods to release the negative emotions generated by the stressor, resulting in bullying behavior (Agnew, 1992). Moreover, poor peer relationships resulting from low self-esteem increase the likelihood of individuals with a history of abuse being rejected and excluded in peer relationships, exacerbating their aggressiveness and risk of becoming aggression victims (Ettekal and Ladd, 2019). Furthermore, due to poor friendships and lack of support or protection from peers, they exhibit a higher degree of victimization (Sentse et al., 2013). Therefore, we hypothesized that self-esteem and peer relationships would mediate the relationships between childhood abuse, bullying (H5a) and bullying victimization (H5b).

#### 2.6.2 The serial mediating role of self-esteem and moral disengagement

Childhood experiences of abuse can severely damage an individual’s self-esteem and reduce their self-efficacy and locus and control (Weindl et al., 2018). Self-esteem reflects the discrepancy between the actual state of the self as perceived by the individual and ideal or expected state (Rosenberg, 1965b). This discrepancy can become a threat to the self. Individuals with low self-esteem are susceptible to experiencing this self-threat. According to the self-regulation theory, when defending against self-threat, individuals often rationalize current behavior (Bandura, 1989), selectively accept information (Pyszczynski et al., 1999), and attribute the source of the self-threat to external rather than internal factors (Lilly and Wipawayangkool, 2017). This is akin to the mechanisms of moral disengagement, such as moral justification, diffusion of responsibility, and displacement of responsibility, indicating that individuals with low self-esteem may frequently apply the mechanisms of moral disengagement, evade responsibility, and have strong moral disengagement (Zhou et al., 2023). The predictive role of moral disengagement for destructive behaviors, such as aggression and bullying, is well established (Bjärehed et al., 2019a; Thornberg, 2023).

The cycle of violence that begins with childhood abuse may be realized through the decline in self-esteem and cognitive mechanism of moral disengagement, evolving into bullying behavior. Our hypothesis (H6a) proposes that self-esteem and moral disengagement mediate the effects of the relationship between childhood abuse and bullying.

Moral disengagement combined with learned helplessness suggests that individuals with a history of abuse may use the mechanism of moral disengagement to accept or even regard the bullying they experience as acceptable or justified, thereby reducing internal cognitive dissonance (Peterson and Seligman, 1983). This perpetuates the victim cycle. Moral disengagement also affects defending behavior in bullying contexts; a high frequency of defending is associated with less use of moral disengagement strategies (Bussey et al., 2020). In other words, strong moral disengagement is correlated with a low frequency of defending actions. Although defending behavior in bullying situations often refers to the protection of the victim by others, in reality, the victim’s self-protection is the key factor in avoiding becoming a victim. However, the strategy of moral disengagement makes victims more inclined to abandon self-protection, thus becoming victims again (Wang et al., 2022). Therefore, we hypothesized that self-esteem and moral disengagement would mediate the effects of childhood abuse on bullying victimization (H6b).

#### 2.6.3 The serial mediating role of peer relationships and moral disengagement

According to the attachment theory (Bretherton, 1992), individuals with a history of childhood abuse often struggle to form secure attachment relationships. As attachment is a crucial component of peer relationships, childhood abuse often leads to poor peer interactions. Poor peer interactions can affect an individual’s moral decision-making, moral development, and attribution style (Garrigan et al., 2018). The mechanism of moral disengagement is essentially a form of moral self-regulation that involves a disinhibited attribution style, a form of moral decision-making; therefore, peer relationships may also influence the level of moral disengagement. As noted above, strong moral disengagement positively predicts bullying and can rationalize victimization, causing victims to abandon self-protection and become long-term bullying targets. Therefore, we hypothesized that peer relationships and moral disengagement would mediate the relationships between childhood abuse, bullying (H7a), and bullying victimization (H7b).

#### 2.6.4 The serial mediating role of self-esteem, peer relationships, and moral disengagement

Childhood abuse experiences can lead to low self-esteem. Individuals with low self-esteem struggle to establish secure attachment relationships with peers, resulting in poor peer relationships and a source of stress. This stress is released through moral disengagement in the form of aggressive behavior, leading to bullying. Based on the general aggression model, childhood abuse, self-esteem, peer relationships, and moral disengagement serve as input variables that activate corresponding aggressive schemas, resulting in bullying behavior (DeWall et al., 2011). Furthermore, low self-esteem resulting from an individual’s childhood abuse experiences often causes poor peer relationships, which may cause a failure in moral self-regulation, leading to high levels of moral disengagement. This makes the individual susceptible to rejection and abandoning self-protection, thereby resulting in bullying victimization.

**FIGURE 1:**
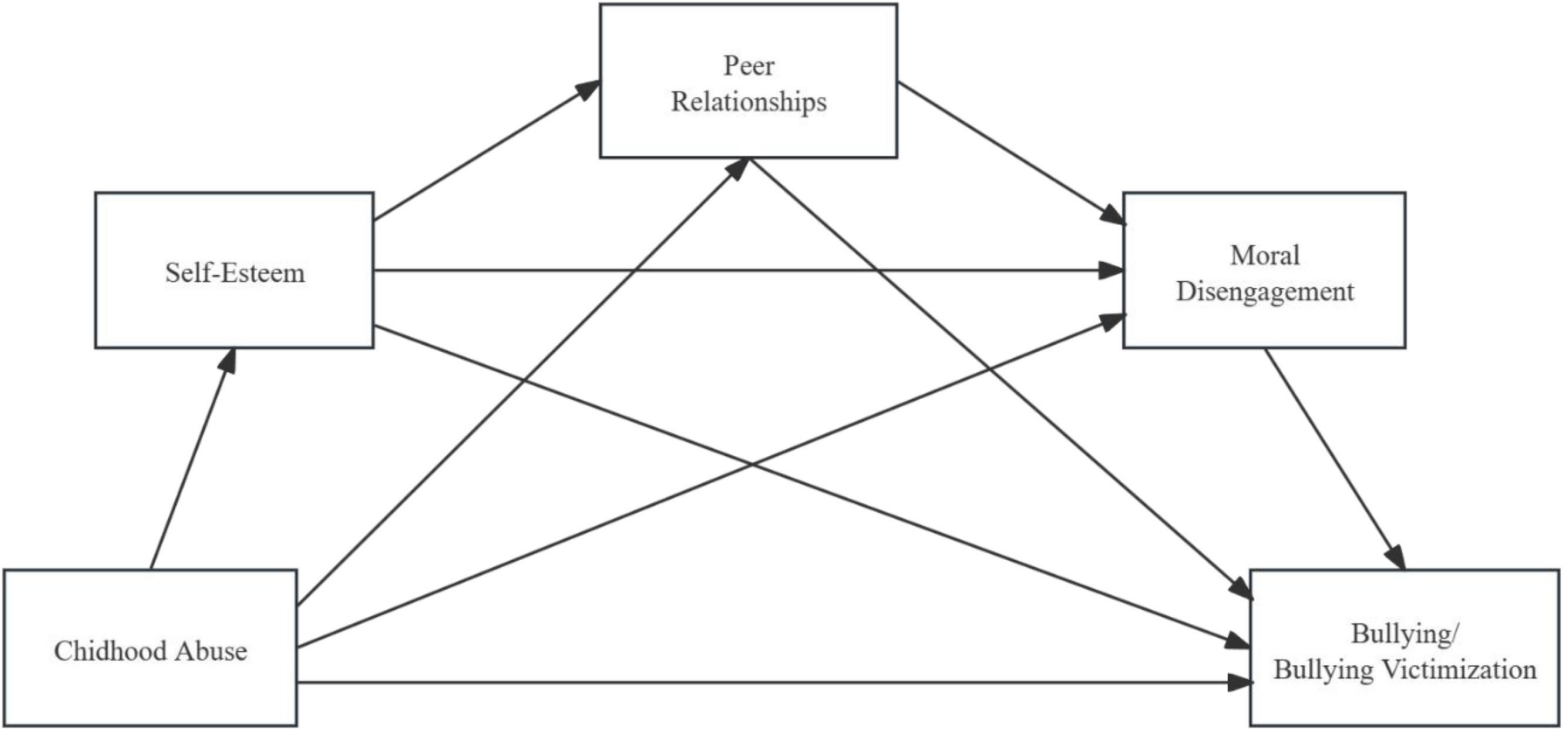
Conceptual models 1 and 2.

Therefore, we hypothesized that self-esteem, peer relationships, and moral disengagement would play chain mediating roles in the relationships between childhood abuse, bullying (H8a), and bullying victimization (H8b). As shown in Figure 1.

## 3 Materials and methods

### 3.1 Participants and procedure

This study conducted a cross-sectional questionnaire survey. We recruited first and second year students from three junior high schools in Sichuan Province, China, totaling 1,548 students. We requested special time from each school and arranged for a questionnaire to be administered to each class individually in their classrooms and at the same time. Participation in the study was voluntary, and all participants provided written informed consent before participating. Students who do not agree to participate in this study will not be given the questionnaire. The questionnaire will be completed anonymously and we guarantee that this survey will be used for research purposes only and the results will be kept strictly confidential. In addition, the study was approved by the relevant research ethics committee. We collected all the questionnaires and then screened and processed them. A total of 1,327 valid questionnaires were obtained after excluding 131 questionnaires that were filled in randomly or omitted. Students from two schools lived in urban areas, whereas students from one school lived in a rural area. The sample comprised 700 women (52.3%) and 627 men (47.3%), including 228 first-year and 1,099 second-year junior high school students.

### 3.2 Measures

#### 3.2.1 Childhood abuse

The Short Form of the Childhood Trauma Questionnaire (CTQ-SF) was used to measure children’s experience of being neglected and abused (Bernstein et al., 2003). The CTQ-SF measures childhood abuse in five dimensions: physical neglect, physical abuse, emotional neglect, emotional abuse, and sexual abuse. It contains 28 items, including five items in each subscale, such as “People in my family hit me so hard it left me with bruises or marks,” and three validity evaluation items. Responses were rated on a five-point Likert scale (1 = never; 5 = very often). Scores on the five subscales were summed, with totals ranging from 25 to 125 and higher scores indicating more severe childhood abuse experiences. The CTQ-SF previously showed good reliability, content validity, and construct validity in the Chinese context (Zhao et al., 2005). In this study, the Cronbach’s alpha coefficient was 0.818.

#### 3.2.2 Bullying and bullying victimization

The Bullying Participant Behaviors Questionnaire (BPBQ) was used to measure bullying behavior. The scale’s reliability and validity were confirmed with a Chinese domestic student population (Demaray et al., 2014; Qiu et al., 2020). This study used the bully (e.g., “I have called another student bad names”) and victim subscales (“I have been called mean names”) of the questionnaire to measure bullying and victimization, respectively. Responses were rated on a five-point Likert scale (1 = never; 5 = six times or more) to describe experiences in the past 30 days, with no reverse scoring questions. The Cronbach’s alpha coefficient of the scale in this study was 0.938.

#### 3.2.3 Self-esteem

The self-esteem scale (SES) was used to measure self-esteem (Rosenberg, 1965a). The scale comprises 10 items, with five scored positively and five scored negatively (e.g., “At times I think I am no good at all”). Responses were rated on a five-point Likert scale (1 = strongly disagree; 4 = strongly agree). The scale exhibited good reliability and validity with secondary school and university students in China (Yan et al., 2021). In this study, the Cronbach’s alpha coefficient was 0.804.

#### 3.2.4 Peer relationships

The Peer Attachment scale was selected from the Inventory of Parent and Peer Attachment (IPPA) developed by Greenberg et al. (1983), which was validated in China (Zhang et al., 2011). The scale comprises 25 items in three dimensions: peer trust, peer communication, and peer alienation. One illustrative item is “I like to get my friend’s point of view on things I’m concerned about.” Responses were rated on a five-point Likert scale (1 = almost never or never true; 5 = almost always or always true). Higher scores indicated a higher degree of attachment to the subject’s peers and better peer relationship. The Cronbach’s alpha coefficient in this study was 0.899.

#### 3.2.5 Moral disengagement

The Moral Disengagement Scale was used to measure the level of moral disengagement (Bandura et al., 1996). The scale comprises 32 items divided into eight dimensions: moral justification, palliative comparison, euphemistic labeling, displacement of responsibility, diffusion of responsibility, minimizing, ignoring, or misconstruing the consequence, dehumanization, and attribution of blame. One illustrative item is “It is alright to fight to protect your friends.” Responses were rated on a five-point Likert scale (1 = strongly disagree; 5 = strongly agree), with higher scores indicating higher levels of individual moral disengagement. The scale had good reliability and validity in the Chinese context (Wang and Yang, 2010). The Cronbach’s alpha coefficient in this study was 0.89.

## 4 Results

### 4.1 Common method bias

A Harman single factor test was used to eliminate common method bias due to the questionnaire method. The results of the factor analysis identified 20 eigen values (unrotated) greater than 1. The amount of variation explained by the first factor was 17.99% (less than 40%), indicating that no significant common method bias was present.

### 4.2 Descriptive statistics

Means, standard deviations, and correlation coefficients of all variables are shown in Table 1.

**TABLE 1:** Descriptive statistics and correlations among variables.

Variable	*M* ± SD	1	2	3	4	5	6
1. Childhood abuse	40.09 ± 13.01	1					
2. Self-esteem	27.99 ± 5.13	−0.517**	1				
3. Peer relationships	87.21 ± 16.22	−0.491**	0.490**	1			
4. Moral disengagement	63.58 ± 15.16	0.368**	−0.280**	−0.266**	1		
5. Bullying	14.93 ± 6.46	0.306**	−0.249**	−0.212**	0.332**	1	
6. Bullying victimization	16.24 ± 9.11	0.404**	−0.369**	−0.311**	0.228**	0.548**	1

**p < 0.01.

### 4.3 Gender and grade

Gender-based homogeneity of variance test showed that the variance of bullying behavior was not aligned; therefore, the Welch *F*-test was used. The results showed that all boys scored significantly higher than girls in school bullying behavior (*F* = 9.38, *p* < 0.05).

Grade-based homogeneity of variance test showed inhomogeneity of variance in bullying behavior; therefore, the Welch *F*-test was used. The results showed that all first-year students had significantly higher bullying behavior scores than second-year students (*F* = 20.55, *p* < 0.001).

### 4.4 Chain mediating roles of self-esteem, peer relationships, and moral disengagement in the relationship between maltreatment and bullying behavior in childhood

The bootstrap technique was used to conduct mediation analysis. SPSS Process Macro 3.0 control prepared by Hayes. Gender and grade were used as the control variables in model 6. The regression results for bullying are presented in Table 2.

**TABLE 2:** Regression analysis results for bullying.

Regression equations							95% CI	
Outcome	Predictors	*R*	*R* ^2^	*F*	B	*t*	Bottom	Top
SE		0.54	0.29	178.73***				
	CA				−0.20	−21.08***	−0.21	−0.18
	Gender				−1.04	−4.34***	−1.50	−0.57
	Grade				1.11	4.55***	0.63	1.59
PS		0.57	0.33	161.14***				
	CA				−0.39	−11.93***	−0.46	−0.33
	SE				0.99	11.66***	0.82	1.15
	Gender				0.79	1.07	−0.66	2.24
	Grade				3.30	4.36***	1.82	4.79
MD		0.40	0.16	49.84***				
	CA				0.32	8.95***	0.25	0.40
	SE				−0.33	−3.55***	−0.51	−0.15
	PS				−0.08	−2.70**	−0.13	−0.02
	Gender				−2.52	−3.26**	−4.04	−1.00
	Grade				1.01	1.26	−0.56	2.58
Bullying		0.41	0.17	44.13				
	CA				0.08	5.13***	0.05	0.11
	SE				−0.13	−3.34***	−0.21	−0.05
	PS				−0.01	−1.03	−0.04	0.01
	MD				0.10	8.45***	0.076	0.12
	Gender				−1.22	−3.71***	−1.87	−0.58
	Grade				0.65	1.92	−0.015	1.32

CA, child abuse; SE, self-esteem; PS, peer relationships; MD, moral disengagement.

**p < 0.01,

***p < 0.001.

Childhood abuse significantly and negatively predicted self-esteem and peer relationships, and significantly and positively predicted level of moral disengagement and bullying behavior. So H1a was supported. Self-esteem significantly and positively predicted peer relationships, and significantly and negatively predicted level of moral disengagement and bullying behavior. Peer relationships significantly and negatively predicted level of moral disengagement. Moral disengagement significantly and positively predicted bullying behavior.

The chain mediation model is presented in Table 3.

**TABLE 3:** Chain mediation model 1.

Type of effect	Path	Effect size	95% CI	
			Bottom	Top
Mediation effect	CA → SE → Bullying	0.0258	0.0084	0.0448
	CA → PS → Bullying	0.0049	−0.0046	0.0149
	CA → MD → Bullying	0.0320	0.0204	0.0448
	CA → SE → PS → Bullying	0.0024	−0.0023	0.0071
	CA → SE → MD → Bullying	0.0064	0.0024	0.0112
	CA → PS → MD → Bullying	0.0030	0.0005	0.0059
	CA → SE → PS → MD → Bullying	0.0015	0.0003	0.0029
Total effect		0.0761	0.0548	0.0981

CA, child abuse; SE, self-esteem; PS, peer relationships; MD, moral disengagement.

Several mediating pathways were identified: CA → SE → Bullying (supporting H2a), CA → MD → Bullying (supporting H4a), CA → SE → MD → Bullying (supporting H6a), CA → PS → MD → Bullying (supporting H7a), CA → SE → PS → MD → Bullying (supporting H8a). The 95% confidence intervals for all five of these paths did not encompass 0, indicating significant indirect effects of the three mediating variables. The 95% confidence intervals for CA → PS → Bullying and CA → SE → PS → Bullying encompass 0, so H3a and H5a do not support.

**FIGURE 2:**
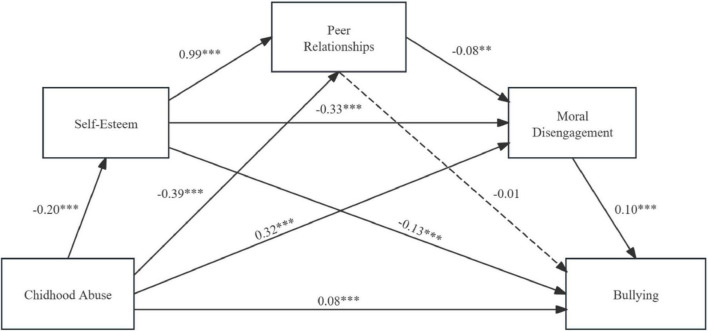
Chain mediation model 1. **p < 0.01, ***p < 0.001.

A serial mediation model was developed, with childhood abuse as the antecedent variable; self-esteem, peer relationships, and moral disengagement as the mediating variables; and bullying as the outcome variable (Figure 2).

### 4.5 Chain mediation of self-esteem, peer relationships, and moral disengagement in the relationship between childhood abuse and bullying victimization

The regression results for bulling victimization are presented in Table 4.

**TABLE 4:** Regression analysis results for bulling victimization.

Regression equations							95% CI	
Outcome	Predictors	*R*	*R* ^2^	*F*	B	*t*	Bottom	Top
SE		0.54	0.29	178.73***				
	CA				−0.20	−21.08***	−0.21	−0.18
	Gender				−1.04	−4.34***	−1.50	−0.57
	Grade				1.11	4.55***	0.63	1.59
PS		0.57	0.33	161.14***				
	CA				−0.39	−11.93***	−0.46	−0.33
	SE				0.99	11.66***	0.82	1.15
	Gender				0.79	1.07	−0.66	2.24
	Grade				3.30	4.36***	1.82	4.79
MD		0.40	0.16	49.84***				
	CA				0.32	8.95***	0.25	0.40
	SE				−0.33	−3.55***	−0.51	−0.15
	PS				−0.08	−2.70**	−0.13	−0.02
	Gender				−2.52	−3.26**	−4.04	−1.00
	Grade				1.01	1.26	−0.56	2.58
Bullying victimization		0.46	0.21	58.45***				
	CA				0.17	7.83***	0.13	0.21
	SE				−0.33	−6.16***	−0.44	−0.23
	PS				−0.04	−2.64**	−0.08	0.01
	MD				0.04	2.25*	0.005	0.07
	Gender				−0.92	−2.04*	−1.81	−0.03
	Grade				−0.47	−1.01	−1.38	0.44

CA, child abuse; SE, self-esteem; PS, peer relationships; MD, moral disengagement.

*p < 0.05,

**p < 0.01,

***p < 0.001.

Childhood abuse significantly and negatively predicted self-esteem and peer relationships, and significantly and positively predicted level of moral disengagement and bullying victimization. So H1b was supported. Self-esteem significantly and positively predicted peer relationships, and significantly and negatively predicted level of moral disengagement and bullying victimization. Peer relationships significantly and negatively predicted level of moral disengagement and bullying victimization. Moral disengagement significantly and positively predicted bullying victimization behavior.

The chain mediation model is presented in Table 5.

**TABLE 5:** Chain mediation of self-esteem, peer relationships, and moral disengagement in the relationship between childhood abuse and bullying victimization.

Type of effect	Paths	Effect size	95% CI	
			Bottom	Top
Mediation effect	CA → SE → Bullying victimization	0.0654	0.0422	0.0909
	CA → PS → Bullying victimization	0.0175	0.0037	0.0324
	CA → MD → Bullying victimization	0.0117	0.0003	0.0237
	CA → SE → PS → Bullying victimization	0.0086	0.0019	0.0157
	CA → SE → MD → Bullying victimization	0.0023	0.0000	0.0056
	CA → PS → MD → Bullying victimization	0.0011	0.0000	0.0030
	CA → SE → PS → MD → Bullying victimization	0.0005	0.0000	0.0014
Total effect		0.1071	0.0787	0.1358

CA, child abuse; SE, self-esteem; PS, peer relationships; MD, moral disengagement.

Several mediating pathways were identified: CA → SE → Bullying victimization (supporting H2b), CA → PS → Bullying victimization (supporting H3b), CA → MD → Bullying victimization (supporting H4b), CA → SE → PS → Bullying victimization (supporting H5b), CA → SE → MD → Bullying victimization (supporting H6b), CA → PS → MD → Bullying victimization (supporting H7b), CA → SE → PS → MD → Bullying victimization (supporting H8b). The 95% confidence intervals for all seven of these paths did not encompass 0, indicating significant intermediate effects of the three mediating variables.

**FIGURE 3:**
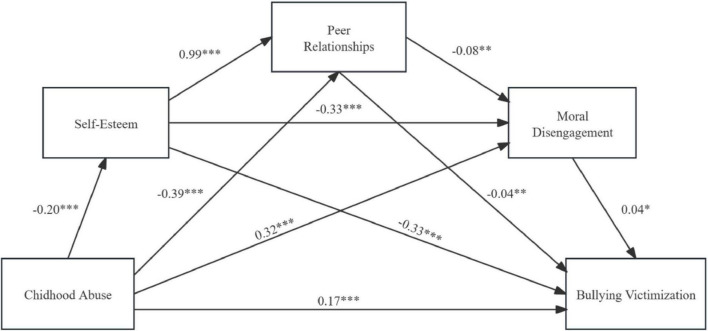
Chain mediation model 2. *p < 0.05, **p < 0.01, ***p < 0.001.

A serial mediation model was developed, with childhood abuse as the antecedent variable; self-esteem, peer relationships, and moral disengagement as the mediating variables; and bullying victimization as the outcome variable (Figure 3).

## 5 Discussion

Childhood abuse and school bullying are serious public health issues with significant negative consequences globally (Chandan et al., 2020). An increasing number of studies have shown a correlation between the two (Li et al., 2024; Wang et al., 2020). However, the impact of cultural factors appears to have led to the neglect of child abuse in China. Influenced by Confucianism, Chinese parents often perceive their offspring as an extension of their own being, akin to private property. They maintain the belief that disciplining their children through physical punishment is not only their prerogative but also a manifestation of their affection, employed as a means to educate and guide, or “Guan,” their young (Wang et al., 2019). This study revealed the chain mediation effects of self-esteem, peer relationships, and moral disengagement on the relationships between childhood abuse, bullying, and bullying victimization. These findings suggest that experiences of childhood abuse may lead to low self-esteem, poor peer relationships, and high levels of moral disengagement. Low self-esteem induces poor quality of peer relationships; poor peer relationships affect the use of moral disengagement mechanisms; ultimately, the moral disengagement mechanism triggers individual behavioral responses in bullying situations. The results revealed positive correlations between childhood abuse, bullying, and bullying victimization. Moreover, self-esteem, peer relationships, and moral disengagement had chain mediating effects on these relationships.

### 5.1 Childhood abuse, bulling, and bullying victimization

Consistent with previous studies, this study found that childhood abuse positively predicted both bullying and bullying victimization. This suggests that children who have suffered abuse are more likely to engage in bullying behavior and become victims of bullying. Childhood abuse is a risk factor for individual bullying and victimization (Jaffee, 2017; Rameckers et al., 2021). Li et al. (2024) demonstrated that childhood abuse was significantly and positively correlated with cyberbullying and victimization. Macalli et al. (2021) found that the experience of childhood abuse increased the likelihood of peer victimization. Experiences of abuse can lead to distrust of others, feelings of powerlessness, and stigmatization, increasing the possibility of individuals developing inappropriate behaviors, such as pathological dependence, substance abuse, and multiple personalities disorder, for survival (Guo et al., 2023). Therefore, the experience of abuse not only increases the likelihood of individuals resorting to inappropriate behaviors, such as violence and bullying, but also makes them susceptible to neglect, rejection, and bullying victimization.

### 5.2 The role of self-esteem

This study demonstrated that self-esteem had significant mediating effects on the relationships between childhood abuse, bullying, and bullying victimization, which was consistent with the findings of previous research (Díaz-Faes and Widom, 2024). According to the attachment theory of self-esteem, children who grow up in environments of abuse and neglect are prone to developing low self-esteem. Low self-esteem generates self-threat and defense, making individuals aggressive and prone to violent behaviors, such as bullying. Moreover, low self-esteem leads to behavioral and emotional problems and a reactive pattern of generalized negative feedback, often making individuals the targets of bullying.

### 5.3 The role of peer relationships

This study revealed that the mediating effect of peer relationships on the association between childhood abuse and bullying was not significant, whereas their mediating effect on the relationship between childhood abuse and bullying victimization was significant. Previous studies have often associated peer relationships with aggressive behaviors (Card et al., 2008); however, aggressive behaviors and bullying are not entirely the same. In addition to various forms of aggression, bullying behavior includes the abuse of power, which is a moral aspect. Peer relationships have the characteristics of dyadic relationships, which can help balance power dynamics in bullying (Prabaharan et al., 2024), thus inhibiting the occurrence of bullying. According to the general aggression model and general strain theory, experiences of abuse and poor peer relationships can make individuals more aggressive, increasing the risk of becoming aggression victims. Furthermore, poor peer relationships can lead to decreased peer support, which makes individuals susceptible to bullying victimization by peers. The absence of friends in school makes children vulnerable (Kendrick et al., 2012).

### 5.4 The role of moral disengagement

This study demonstrated that the mediating effects of moral disengagement on the relationships between childhood abuse, bullying, and bullying victimization were significant, which was consistent with the findings of previous research (Wang et al., 2019; Wang et al., 2022). Bullying involves moral decision-making, and moral disengagement is an important disinhibiting factor for destructive behavior that reduces the guilt and self-blame associated with destructive actions. Therefore, moral disengagement is an internal cognitive mechanism of the effect of childhood abuse on subsequent bullying.

Moral disengagement significantly affects protective behaviors in bullying situations (Bussey et al., 2020). When individuals are inclined to morally disengage, they tend to show less resistance to bullying behavior, leading to bullying victims finding it difficult to receive protection from others when under attack. Moreover, moral disengagement causes individuals to be unable to recognize abuse when they are victims (Cuadrado-Gordillo et al., 2020). In other words, moral disengagement changes an individual’s perception of victimization and regulates the acceptance of violent behavior. This causes individuals to ignore or condone bullying behavior when faced with it, leading to bullying victimization. In addition, Marín-López et al. (2020) reported that strong moral disengagement was associated with a high degree of involvement in cyberbullying, indicating that individuals also face a high risk of victimization online.

### 5.5 The chain mediation

The results revealed chain mediating effects of self-esteem, peer relationships, and moral disengagement on the relationships between childhood abuse, bullying, and bullying victimization. This finding integrated environmental, personality, and cognitive factors into a unified path model, enhancing research on the mechanism causing bullying and bullying victimization. The findings of this study could help develop interventions to prevent bullying and bullying victimization.

Based on the general aggression model, childhood abuse, self-esteem, peer relationships, and moral disengagement form a chain of input that ultimately realizes the cycle of violence from abusive experiences to bullying behavior. Experiences of childhood abuse can lower an individual’s self-esteem (Arslan, 2016; Díaz-Faes and Widom, 2024). Low self-esteem affects the formation of peer attachment, leading to poor peer relationships. Poor peer interactions can impact an individual’s moral development and attribution style (Garrigan et al., 2018). Furthermore, poor peer relationships make individuals susceptible to peer rejection, and peer rejection may activate the process of moral disengagement (Fontaine et al., 2012), resulting in a high level of moral disengagement. A high level of moral disengagement is positively correlated with bullying behavior (Bjärehed et al., 2019b; Romera et al., 2021). Therefore, this study identified the chain mediating effect of self-esteem, peer relationships, and moral disengagement between childhood abuse and bullying.

Based on the developmental cascades theory, the impact of abuse experiences is transmitted through multiple factors and levels in the individual’s developmental process, forming a cycle of victimization (Li and Xia, 2024). The effects of abuse experiences permeate into moral development through self-esteem and peer relationships (Park et al., 2017), thereby affecting the level of moral disengagement, reducing the self-protection of potential bullying targets, and leading to bullying victimization.

### 5.6 Implications and limitations

School bullying is a widespread and contemporary social issue that deserves attention. Although we could not obtain definitive answers through a cross-sectional survey, this study shed light on the negative impacts of childhood abuse, indicating that parents should focus on the physical and mental health of their children. The chain mediating pathways highlighted the roles of personality, peer, and cognitive factors in the cycles of violence and victimization. This provides suggestions for developing interventions to mitigate the negative impacts of childhood abuse and prevent bullying and bullying victimization. The key to breaking the cycles of violence and victimization may lie in reconstructing a family-centered moral development framework for youth, consolidating a holistic cultivation approach that prioritizes both character and competence, coordinates intellectual growth with emotional maturity, and upholds “cultivated refinement” as the primary evaluation criterion, while avoiding the formation of success paradigms measured solely by physical dominance or competitive outcomes.

This study had several limitations. First, we did not examine the patterns of bullying and bullying victimization associated with childhood abuse to determine whether it has a stronger correlation with subsequent bullying, bullying victimization, or both. Second, we could not determine whether childhood abuse was associated with instrumental or reactive bullying. Third, all variables were measured with self-report scales, which may lead to the participant response bias. Finally, as this was a cross-sectional study, we could not draw inferences of causal relationships. Future research should further investigate these issues with longitudinal data.

## 6 Conclusion

The results of this study suggest that the adverse effects of childhood abuse on adolescent bullying behavior and bullying victimization require more attention of governments and teachers. Our findings indicate that childhood abuse may reduce self-esteem, affecting their peer relationships. Moreover, moral disengagement promotes bullying behavior. Our study highlights the importance of childhood abuse experiences, self-esteem, peer relationships, and moral disengagement in understanding adolescent bullying behavior. These findings enhance our understanding of adolescent behavior and psychology and provide a theoretical basis for developing interventions to prevent negative behaviors in schools.

## Data Availability

The datasets presented in this study can be found in online repositories. The names of the repository/repositories and accession number(s) can be found below: https://data.mendeley.com/datasets/ngrb4yx7p6/1. CGJung (2025), “Examining the interplay of childhood abuse, bullying, and bullying victimization in middle school students,” Mendeley Data, V1, doi: 10.17632/ngrb4yx7p6.1.
